# Fecal calprotectin in healthy children aged 4–16 years

**DOI:** 10.1038/s41598-020-77625-7

**Published:** 2020-11-25

**Authors:** María Roca, Ana Rodriguez Varela, Eva Carvajal, Ester Donat, Francisco Cano, Ana Armisen, Maria Jose Vaya, Helena Ekoff, David Hervas, Niclas Rydell, Carmen Ribes-Koninckx

**Affiliations:** 1 Celiac Disease and Digestive Immunopathology Unit, Instituto de Investigación Sanitaria La Fe, P.O. Box 46026, Valencia, Spain; 2 Primary Health Care Center of Betera, P.O. Box 46117, Bétera, Valencia Spain; 3 Department of Paediatrics, Hospital Casa de Salud, P.O. Box 46021, Valencia, Spain; 4 Pediatric Gastrohepatology Unit, Hospital Universitario y Politécnico La Fe, P.O. Box 46026, Valencia, Spain; 5 Thermo Fisher Scientific, PO Box 6460, Uppsala, Sweden; 6 Biostatistics Unit, Instituto de Investigación Sanitaria La Fe, P.O. Box 46026, Valencia, Spain

**Keywords:** Biomarkers, Gastroenterology

## Abstract

Reference values of fecal calprotectin (fCP) have not been convincingly established in children. We aimed to investigate fCP concentrations in a larger population of healthy children aged 4–16 years to analyze more in depth the behavior of fCP in this age range and to determine if cut-off levels could be conclusively recommended. A prospective study was conducted to investigate fCP concentrations of healthy children aged 4–16 years. In 212 healthy children, the median and 95th percentile for fCP were 18.8 mg/kg and 104.5 mg/kg, respectively. We found a statistically significant association between the 95th percentile of fCP concentrations and age (p < 0.001). We propose a nomogram to facilitate the interpretation of fCP results in children aged 4–16 years. Further studies are required to validate the proposed values in clinical practice.

## Introduction

Calprotectin is a 36 kDa member of the S100 family proteins found in the cytosolic fluid of neutrophils, monocytes, and macrophages^1^. Calprotectin is released following activation of leukocytes, and its ability to inhibit zinc-dependent enzyme systems confers its antimicrobial effects^2^. The altered expression of the S100 family proteins has been shown to play a key role in neurodegenerative and inflammatory disorders^3^. This protein is present in various body fluids and its concentration in feces is about six times that of plasma^4^.

Increased levels of calprotectin in serum, saliva, sputum and feces have been described in various pathological conditions^5–7^ and thus calprotectin measurement could be a valuable biomarker with potential clinical applications. Of all the above, feces samples are the most commonly used in clinical practice, especially in the diagnosis and management of Inflamatory Bowel Disease (IBD). In addition, it is relatively resistant to enzymatic degradation; the literature showing that fCP remains stable at room temperature between 3 and 7 days^4,8,9^.

The fCP has been recognized as a non-invasive biomarker of gastrointestinal inflammation^10^. Various factors influence fCP levels, especially age but also medication, and daily variation^11–13^. Additionally, fCP results vary depending on the methods and commercial kits used for its measurement and reference values of fCP have not been convincingly established in children.

In our previous work on a healthy population of 174 children aged 0–12 years, we found that young children had higher fCP concentrations compared to adults^14^. The highest concentration of fCP was found in infants younger than 4 years of age, but with large individual variations^14^. A tendency towards less variation in fCP concentrations was observed from 4 years onwards, however a convincingly clear cut off value for children aged 4 and older was difficult to establish.

Also, other authors found fCP values to be lower and with less variability and approaching reference values for adults, in children older than 4 as compared to younger children (0–4 years). However, due to the diversity of published results there is currently no consensus on fCP reference values for pediatric patients.

The principal clinical utility of fCP at present is in the diagnosis and monitoring of IBD as it is reported to be a better screening tool for the presence of IBD in undiagnosed patients than blood inflammatory markers such as CRP (C reactive protein) or erythrocyte sedimentation rate (ESR). Although it helps in selecting patients in whom IBD needs to be evaluated, it has some limitations as it cannot differentiate ulcerative colitis from Crohn’s disease and there is also no linear correlation between calprotectin levels and the severity or extent of mucosal inflammation, fCP levels cannot predict the therapy outcome either^15^. Measuring fCP repeatedly may be useful in IBD patients with minor or absent clinical symptoms to confirm remission^16^ but also for suspected relapse and to consider a re-evaluation or change of management^17–20^.

The fCP may be considered as a valuable tool to differentiate functional gastrointestinal disorders from IBD. Functional disorders are more prevalent in children than in infants. We therefore, aimed to investigate fCP concentrations in a larger population of healthy children aged 4–16 years to analyze more in depth the behavior of fCP in this age range and to determine if cut-off levels could be conclusively recommended. Knowing the normal range will allow us to make decisions for clinical intervention.

## Materials and methods

### Patients and samples

From January 2015 till December 2019, stool samples from healthy children aged 4–16 years were prospectively collected at the Primary Health Care Center of Betera, Casa de La Salud Hospital and from the Pediatric department of La Fe Hospital. Recruited children were invited to participate when attending Primary Health care routine scheduled visits (Spanish National Health Service protocol) and all met the following inclusion criteria: no illnesses or vaccines in the prior month to enrollment, no hospital admissions 3 months prior to enrollment and no underlying chronic inflammatory disease. The exclusion criteria were the following: any intake of steroidal or non-steroidal anti-inflammatory drugs, gastric acidity inhibitors, antibiotics or any other drug during the 2 weeks prior to recruitment or a history of signs or symptoms of infection or gastrointestinal disease (diarrhea, vomiting, hematochezia, fever).

### Methods

Sample collection and storage was performed according to a previously reported protocol^14^. Briefly, parents were instructed to collect a small amount of feces of one stool passed at any time of the day (one sample per child). The containers with the stool samples were kept in the fridge at home and brought to the laboratory no later than 3 days after collection; samples were then stored at − 20 °C until analysis. For the protein extraction, the fecal sample preparation kit (Roche Diagnostics, Rotkreuz, Switzerland) was used according to the manufacturer’s instructions. Measurement methods were previously described in detail^14^. For fCP concentrations the EliA Calprotectin 2 assay (Thermo Fisher Scientific, Uppsala, Sweden) was used. Concentrations of fCP were expressed as milligrams per kilogram of feces.

### Statistical methods

We estimated both the median (50th percentile) and the 95th percentile with a quantile regression model including a restricted cubic spline for the relationship between fCP concentrations and age. To ease the interpretation of results, a nomogram based on the predictions of the models was developed for both the percentiles. A Monte Carlo simulation was used to estimate the uncertainty in the determination of the 95th percentile and the median for different sample sizes. A sample size of around 200, with a confidence interval length of ± 10 in the worst case scenario (95th percentile), was considered adequate. P values < 0.05 were considered statistically significant. All statistical analyses were performed using R (version 3.6.2). Reference values proposed were calculated based on the lowest 95th percentile.

### Ethical considerations

This study was performed with approval from the Ethics Committee of the University and Polytechnic La Fe Hospital, Valencia, Spain (Approval N. 2014/0157). The data are anonymous, and written informed consent was obtained from the parents as well as from children older than 12 years who participated in the study. This study was conducted per the Helsinki Declaration. All experiments were performed in accordance with relevant guidelines and regulations.

## Results

**Figure 1:**
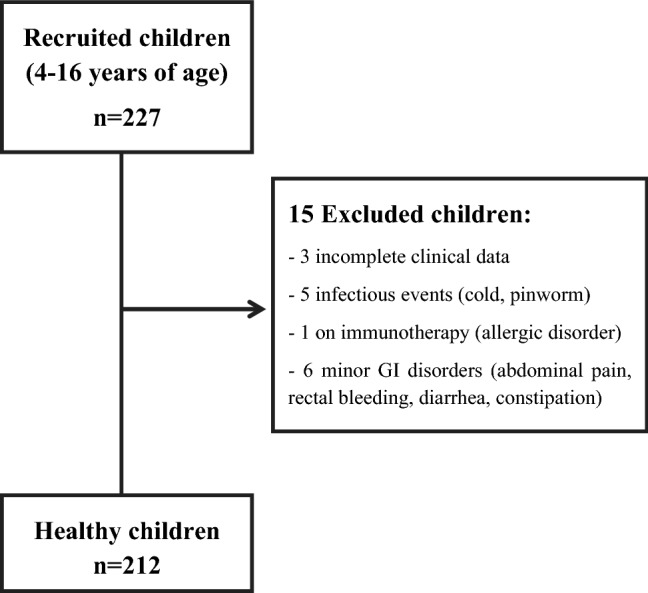
Flowchart.

227 children were recruited. Of these, 15 were excluded from final evaluation due to different reasons (Fig. 1). Finally, 212 (104 girls) healthy children fulfilled the inclusion criteria and were considered for the final analyses. The median age of all participants was 9.2 years, first and third quartiles being 6.2 and 11.5 years, respectively (Table 1).

**Table 1:** Characteristics of the study population.

Age range (years)	n	Age median (1st, 3rd quartiles) (years)	Gender male/female n (%)
4–16	212	9.2 (6.2, 11.5)	108 (50.9)/104 (49.1%)
4 to < 9	100	6.2 (5.2, 7.6)	50 (50%)/50 (50%)
9–16	112	11.5 (10.3, 12.4)	58 (51.8%)/54 (48.2%)

**Figure 2:**
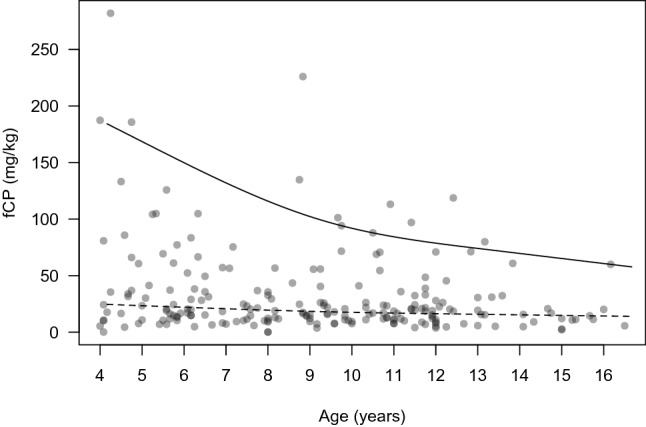
Scatter plot depicting the relationship between age in years and fCP concentrations. Regression lines have been added for the 95th percentile (solid line) and for the 50th percentile (dashed line). (*fCP* fecal calprotectin).

The median of fCP concentrations in the 212 children was 18.8 mg/kg and ranged from 24.8 mg/kg at 4 years to 14.4 mg/kg at 16 years. As shown in Fig. 2, the median values decreased gradually depicting a smooth slope throughout the studied age range. We found no evidence of association between the median fCP concentration and age (p = 0.26) or gender (p = 0.8).

The 95th percentile of fCP concentrations for the whole group was 104.5 mg/kg and ranged from 187.4 mg/kg at 4 years to 60.7 mg/kg at 16 years. We found a statistically significant association between the 95th percentile of fCP concentrations with age (p < 0.001), and this association was stronger at younger ages and decreased at around 9 years of age (Fig. 2). As shown in Fig. 2, a steeper slope was observed in the 95th percentile levels in children from 4 to 9 years of age, due to a greater dispersion of the fCP values. For children older than 9–10 years, a gentler slope was observed, related to less variability in fCP values at older ages.

**Figure 3:**
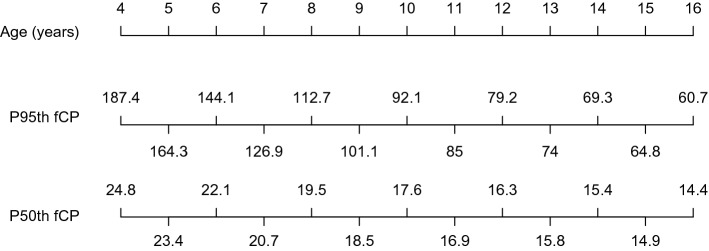
Nomogram of healthy children (4–16 years of age) for the 95th and 50th percentile of fCP levels. (*fCP* fecal calprotectin). The nomogram comprises three rows: the first shows the age in years of healthy children from 4 to 16, the second row shows the 95th percentile of the fCP concentrations and the third row, the median of the fCP concentrations.

Additionally, we have developed a nomogram based on the results obtained (Fig. 3). The figure shows the age of the children in years, and the 95th and 50th percentile of fCP levels.

## Discussion

We have evaluated the concentrations of fCP in healthy children aged 4–16 years and for the first time, we propose a nomogram to facilitate the interpretation of fCP results in this age range. In the youngest children, we found higher fCP values for the 95th percentile and more variability in fCP concentrations than in older children; consequently, we observed a steeper slope from 4 to 9 years and a smoother decline in the 95th percentile and more steady values from 9 to 16 years. Therefore, it is difficult to establish a unique fCP cut-off in children for the whole age range, and thus we propose a nomogram that enables estimation of the fCP levels in children according to their age in years.

Although the median concentration of fCP for the whole study population, i.e. 18.8 mg/kg, was below the cut-off value of 50 mg/kg proposed for adults^8^, about 20% of our healthy children had fCP concentrations above 50 mg/kg. Moreover, the 95th percentile of fCP concentrations, i.e. 104.5 mg/kg, obtained for the age range of 4–16 years was higher than the recommended cut-off value for adults.

Different concentrations of fCP have been published for healthy children of the same age range as in our study; this variability being partially related to the use of different commercial kits and methods^21^. The median fCP values for children older than 4 years in previous publications^14,22–24^ were below 50 mg/kg, as in our study. However, Davidson et al.^25^ reported a median fCP slightly higher (62 mg/kg) for a population aged 4–17.9 years.

Moreover, some studies report 95th or 97.5th percentile concentrations and/or recommended cut-off values^14,22,25,26^. Fargerber et al.^22^ suggested that the cut-off level for adults (50 mg/kg) could also be used for children older than 4 years regardless of gender, based on the fCP 95th percentile found in their study (43.4 mg/kg). Davidson et al.^25^ defined an upper normal limit of fCP of 62 mg/kg for the age group of 4–17.9 years and proposed that this fCP cut-off could be used for the diagnosis of IBD in both adults and children over the age of 4. In another prospective study^26^ the authors suggested age-related reference ranges (calculated as the 97.5th percentile) for fCP: 2–9 years < 166 mg/kg and 10–59 years < 51 mg/kg.

In our previous study^14^ we showed a nomogram with the 95th and 50th percentiles of fCP values for children 0–12 years of age. In the present work, by expanding the sample size of children from 4 to 16 years (which is the limit of the pediatric age in many countries), we have been able to provide a new nomogram focusing on this age range.

As explained above, previous published studies on the values of fCP in healthy children were heterogeneous, from several perspectives: the used method for fCP analysis as well as the performed statistical analyses were different, the subjects were gathered into different age groups based on diverse criteria, and in addition, many of these works presented data from the median values but not from the 97th/95th percentiles. For all these reasons, not only comparison of the studies is difficult, but also inferring general reference values is extremely challenging. Moreover, due to the variability of fCP concentrations, especially in younger children, establishing a unique reference value for the pediatric population is not feasible. However, if we consider the lowest value of the 95th percentile for children from 4 to 16 years of age, we obtain a slightly higher reference value for fCP (i.e. 61 mg/kg), as compared to the 50 mg/kg cut-off established for adults. By selecting the lowest value of the 95th percentile instead of the median (i.e. 18.8 mg/kg) to establish reference values, we reduce false positive results, thus avoiding unnecessary additional evaluations. On the other hand, if we select the highest 95th percentile in the entire age range (4–16 years) as reference value (i.e. 187 mg/kg), we would be limiting even more the number of false positive results while keeping the risk for false negatives at this cut-off still very low, as levels higher than 250 mg/kg are usually being reported in active IBD^20,27^. However, a single reference value for the entire age range cannot be easily established because the values are not uniform enough. Even so, there are no robust and objective criteria to establish different reference values according to different age groups. Therefore, we propose using the nomogram as the most cautious approach.

The fCP is a marker of intestinal inflammation, the detection of which is relatively simple and non-invasive. This makes it a potential tool for helping clinicians to decide on the need for further invasive diagnostic tests. For this reason, having reliable reference values is necessary. Based on our results, in which a non-linear downward trend of the 95th percentile values was observed (which was more pronounced in children from 4 to 9 years old), we suggest the use of nomograms to interpret fCP levels in children from 4 to 16 years. This suggestion based on a sample from a Spanish population should be adapted to the particularities of different populations.

In conclusion, we have developed a useful nomogram for interpretation of fCP levels in children from 4 to 16 years of age. Thus, our present work goes beyond our previous study^14^, adding new and valuable information in an extremely interesting population from the clinical point of view, namely the population ranging from 4 to 16 years, where biomarkers of intestinal inflammation are the most needed in clinical practice. Further studies are required to validate the proposed nomogram approach for interpretation of fCP values in clinical practice.

## Data Availability

Datasets from this study are available from the corresponding author on reasonable request.
